# Clinical Outcomes in Patients Undergoing Anterior Cruciate Ligament Reconstruction With Controlled Accelerated Rehabilitation

**DOI:** 10.7759/cureus.57761

**Published:** 2024-04-07

**Authors:** Ihsan Ozdamar

**Affiliations:** 1 Orthopaedics and Trauma, Marmara University Pendik Training and Research Hospital, Istanbul, TUR

**Keywords:** rehabilitation, hamstring autograft, transfemoral cross-pin, endobutton, anterior cruciate ligament (acl) reconstruction

## Abstract

Background

An anterior cruciate ligament (ACL) tear is one of the most common sports injuries in the knee region. Currently, anatomical ACL reconstruction with quadrupled hamstring tendon autograft and controlled accelerated rehabilitation is a frequently used treatment approach. This study aims to add to the literature the early and mid-term clinical results of the controlled accelerated rehabilitation program we implemented in our clinic to enable patients to return to their daily activities faster after ACL reconstruction.

Methodology

In this retrospective study, 51 patients (50 males, 1 female) diagnosed with ACL tear and undergoing ACL reconstruction with quadrupled hamstring tendon graft were included in our study. In the femoral fixation of the graft, the transfix method was used in 22 patients and the Endobutton-CL method was used in 29 patients. A controlled accelerated rehabilitation program developed by Shelbourne was employed with some modifications to the patients. Clinical evaluation of patients was performed using Lysholm, Cincinnati, Tegner, and International Knee Documentation Committee (IKDC) scoring systems.

Results

The mean postoperative follow-up period was 18.7 months (range = 6-36 months). During the physical examination, Lachman, anterior drawer, and pivot shift, 49 (96%) patients achieved excellent or good results, while only two (5%) patients experienced fair clinical outcomes. On clinical assessment using the Lysholm and IKDC scoring systems, 50 (98%) patients demonstrated excellent or good results, whereas only one (2%) patient showed fair results. Excellent and good outcomes were observed in all patients using the Cincinnati scoring system. We found a significant decrease in Tegner activity score pre-surgery, which significantly increased post-surgery.

Conclusions

The combination of Endobutton and cross-pin for femoral fixation and staples and interference screw for tibial fixation is thought to be safe in patients undergoing controlled accelerated rehabilitation after ACL reconstruction. The implementation of controlled accelerated rehabilitation enables patients to return to their social lives earlier without resulting in clinically poor outcomes in grafts and implants applied in the early and middle stages.

## Introduction

An anterior cruciate ligament (ACL) tear is one of the most common ligament injuries in the knee, occurring at a frequency of 1 in 3,000 in the general population. More than 150,000 anterior cruciate ligament injuries occur each year in the United States. Approximately 50% of these injuries are caused by sports-related incidents, with high incidences observed in sports such as handball, basketball, football, and skiing which involve pivoting movements. The risk of ACL injury in female athletes is twice as high as in male athletes [1-4].

However, despite the numerous studies reported in the literature, the gold standard for graft selection, femoral and tibial fixation material selection, and the rehabilitation protocols to be applied are still not clearly defined [5-7]. In this surgical technique, the hamstring tendon is the most commonly preferred graft, followed by the patellar tendon and quadriceps tendon as other frequently chosen graft types. The most commonly used materials for femoral fixation are Endobuttons, cross-pins, and bioabsorbable screws, while for tibial fixation, bioabsorbable or titanium screws and staples are typically utilized [8,9].

One of the most important factors affecting the outcomes of ACL reconstruction is rehabilitation. While rehabilitating after ACL reconstruction, it is crucial to consider the graft type, how it is secured in the bone tunnels, as well as the patients’ ages (chronological and physiological), activity levels, athletic history, and expectations for future participation [10]. Both standard and accelerated rehabilitation have been described in the literature and their advantages and disadvantages have been reported [9-15]. Standard rehabilitation may result in a delayed range of motion and may make patients more susceptible to possible complications such as arthrofibrosis. The most important feature of accelerated rehabilitation is the early restoration of range of motion and early return to normal life. Although accelerated rehabilitation may be associated with possible tunnel enlargement, decreased graft tone, and stability problems in theory, many clinical studies have confirmed no stability problems after accelerated rehabilitation [12-15]. In our clinic, we utilized the updated version of the controlled accelerated rehabilitation program, as described by Shelbourne in 1992 [11]. This four-phase program involves the development of muscle strength through hamstring and quadriceps tendon stretching exercises during the pre-reconstruction period. Isotonic and isometric exercises are then initiated within the first two weeks after reconstruction. Closed and open kinetic chain exercises, as well as squatting exercises, are introduced starting from the third week, as tolerated. Our rehabilitation program gradually increases knee flexion until the sixth week. Return to sports is not permitted before the sixth month.

This study aims to add to the literature the early and mid-term clinical results of the controlled accelerated rehabilitation program we implemented in our clinic to enable patients to return to their daily activities faster after ACL reconstruction.

## Materials and methods

Study design

In this retrospective study, 65 patients diagnosed with ACL tear and undergoing ACL reconstruction at the Orthopaedics and Traumatology Clinic between 2007 and 2010 were included. The study excluded patients younger than 18 years of age (three patients) and those who underwent meniscus repair (seven patients) and presented with additional knee ligament injuries apart from ACL (four patients), as controlled accelerated rehabilitation protocols could not be effectively implemented in such cases. Finally, 51 patients were analyzed in the study.

Surgical technique

Our patients underwent arthroscopic single-tunnel ACL reconstruction utilizing a quadrupled hamstring tendon graft as the chosen surgical technique. In the femoral fixation of the graft, the transfix method was used in 22 patients, and the endobutton-CL method was used in 29 patients. All patients underwent tibial fixation using absorbable interference screws and staples.

Rehabilitation program

All patients underwent the controlled accelerated rehabilitation program described by Shelbourne et al. with some modifications before and after reconstruction [11].

Phase 1 (pre-reconstruction period) attempted to establish the pre-trauma joint range of motion. Hamstring and quadriceps tendon stretching exercises were performed.

In phase 2 (first two weeks post-reconstruction), all patients were allowed full weight-bearing walking, isotonic and isometric exercises were initiated, attempts were made to achieve 0-degree extension and 90-degree flexion of knee joint movements, and a knee brace was not used.

Phase 3 (three to six weeks post-reconstruction) attempted to increase knee flexion by 10 degrees each week, weighted hamstring and quadriceps stretching exercises were performed, closed and open kinetic chain exercises were initiated, squatting exercises were started in tolerable patients, and walking exercises in the pool were started.

In phase 4 (six weeks to six months post-reconstruction), patients started straight running, proprioception exercises were initiated, bicycle exercises were started, patients returned to their preoperative social lives, and attempts were made to return to sports activities at six months.

As mentioned before, there are some possible complications of accelerated rehabilitation, such as tunnel enlargement, decreased graft tone, and stability problems [12-15]. These complications were taken into consideration in all patients included in the study. In our clinic, not only the surgical complications but also the rehabilitation-related complications were mentioned during the preoperative written informed consent. Patients were included in this rehabilitation after they provided written informed consent.

Study variables

Patients were called for follow-up appointments at one, two, four, six, twelve weeks, and six months post-surgery and annually thereafter. During physical examinations, pain, swelling, range of motion, patellofemoral pain, crepitus, Lachman test, pivot-shift test, and anterior drawer test were performed, and the results were recorded. Regarding physical examinations, Lachman, anterior drawer, pivot shift, and endpoint tests were performed, and results were evaluated as 0-5 mm (+), 6-10 mm (++), and 11-15 mm (+++). Thigh circumference measurements were taken 15 cm proximal to the upper edge of the patella, and the difference between the operated and non-operated sides was recorded. Functional status and satisfaction with surgery were evaluated using the International Knee Documentation Committee (IKDC), Lysholm, Modified Cincinnati, and Tegner scoring systems.

Statistical analysis

Statistical analysis was performed using SPSS Program version 26.0 (IBM Corp., Armonk, NY, USA). The conformity of the variables to normal distribution was examined by visual (histogram and probability plots) and analytical (Kolmogorov-Smirnov test) methods. Descriptive statistics were expressed as mean, standard deviation, and minimum-maximum values for normally distributed variables; median, interquartile range, and minimum-maximum values for non-normally distributed variables; and percentage frequency values for categorical data. For normally distributed variables, the t-test was used for comparison, whereas for datasets conforming to skewed distribution, the Wilcoxon signed-rank Test and Mann-Whitney U test were used. The chi-square test was used to compare categorical data and Fisher’s exact test was used when the chi-square assumption was not met. Statistical significance was considered significant when the p-value was below 0.05.

## Results

**Figure 1:**
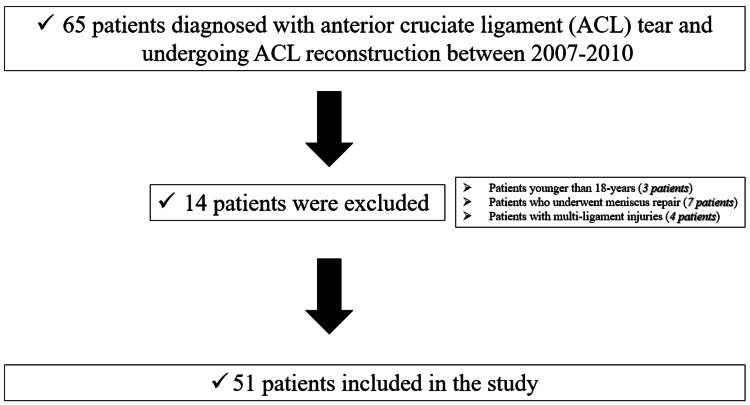
Flowchart of the study groups. Detailed description of included and excluded patients.

A total of 51 patients (50 males, 1 female) were analyzed in the study (Figure 1). The mean age of our patients was 28.8 years (range = 18-42 years), and the mean interval between trauma and surgery was 21.5 months (range = 1-120 months). Diagnostic arthroscopy performed during reconstruction revealed isolated ACL tear in 22 (44%) patients, medial meniscus injury in 18 (36%) patients, lateral meniscus injury in seven (14%) patients, both medial and lateral meniscus tear in one (2%) patient, medial femoral condropathy in five (10%) patients, and intra-articular loose body in one (2%) patients. Partial meniscectomy was performed on 26 (52%) patients with meniscal tears.

Postoperative joint range of motion was measured with a goniometer, and no limitation of extension was observed in any patient. The mean flexion degree was found to be 133 (range = 120-140 degrees). Regarding thigh atrophy, 36 (71%) patients had 0-5 mm atrophy, 10 (19%) patients had 5-10 mm atrophy, three (6%) patients had 10-15 mm atrophy, and two (4%) patients had 15-20 mm atrophy. Patients were asked to jump forward as far as they could on one leg, which was done three times for each knee, comparing the measurements between the operated and non-operated sides. In 96% of patients, values of over 85% were reached, while two patients were between 75-85%. No patient had values below 75%.

During physical examinations, Lachman, anterior drawer, and pivot-shift tests were performed endpoint sensation was soft in two (4%) patients and firm in 49 (95%) patients (Table 1).

**Table 1:** ACL stability test results. ACL = anterior cruciate ligament

Stability tests (N = 51)	(-)	(+)	(++)	(+++)
Lachman	31 (61%)	18 (35%)	2 (4%)	0
Anterior drawer	0	49 (96%)	2 (4%)	0
Pivot shift	35 (69%)	14 (27%)	2 (4%)	0

In functional evaluation, an excellent result was obtained in 38 (76%) patients according to the Lysholm score, and no poor result was encountered in any patient. The mean Lysholm score was 95 (range = 72-100) (Table 2).

**Table 2:** Lysholm scores of the patients.

Lysholm score	Number of patients (N = 51)	Results
95–100	38	Excellent
85–94	12	Good
65–84	1	Fair
64 and under	0	Poor

According to the Modified Cincinnati score, an excellent result was obtained in 44 (88%) patients, and no poor result was encountered in any patient. The Modified Cincinnati scoring system was 28 (range = 22-30) (Table 3).

**Table 3:** Modified Cincinnati scores of the patients.

Modified Cincinnati score	Number of patients (N = 51)	Result
26–30	44	Excellent
21–25	7	Good
16–20	0	Fair
15 and under	0	Poor

Patients’ pre-injury, preoperative, and postoperative activity levels were evaluated using the Tegner Activity Score. Statistical analysis showed a significant decrease in the Tegner Activity Score after injury and a significant increase after surgery (p < 0.0001) (Table 4).

**Table 4:** Descriptive statistics of Tegner activity scores of the patients. The statistical evaluation was performed using the paired t-test based on two-way p-values. SD = estimate of the variability of observations; SEM = estimate of the variability of possible values of means of samples

Tegner activity score	Pre-injury	Preoperative	Postoperative
Mean	6.94	3.78	6.74
SD	0.98	0.96	1.35
SEM	0.13	0.13	0.18
P	<0.0001		

According to the IKDC scoring system, 32 (63%) patients were able to return to normal life and 17 (33%) patients were able to return to near-normal life. While 44 (86%) patients reported intense activity levels pre-injury, this activity level could be reached only in 33 (65%) patients postoperatively. On the other hand, 35 (69%) patients reported sedentary activity levels between the injury and surgery, whereas no patient reported sedentary activity levels in the postoperative period, just as in the pre-injury period. The IKDC scores and activity rates of the patients can be observed in detail in Table 5 and Table 6.

**Table 5:** IKDC scoring ratios. IKDC = International Knee Documentation Committee

IKDC scoring system	Number of patients (N = 51)	Ratio (%)
A (normal)	32	63%
B (near normal)	17	33%
C (abnormal)	2	4%
D (poor)	0	0%

**Table 6:** IKDC activity rates. IKDC = International Knee Documentation Committee

Activity level	Pre-trauma	Preoperative	Postoperative
Level 1 (intense)	44 (86%)	0	33 (65%)
Level 2 (moderate)	4 (8%)	0	12 (23%)
Level 3 (low)	3 (6%)	16 (31%)	6 (12%)
Level 4 (sedentary)	0	35 (69%)	0

With a mean follow-up of 18.7 months (range = 6-36 months), none of the patients experienced a sensation of instability or re-rupture. Skin irritation at the proximal tibia due to implants used for distal graft fixation during reconstruction was observed in 13 (26%) patients, but it did not interfere with daily activities. Mild numbness on the anterior surface of the leg due to damage to the infrapatellar branch of the saphenous nerve during harvesting of the hamstring graft was reported in 14 (28%) patients. One (1.96%) patients developed probable femoral nerve palsy due to a possible tourniquet-related issue during reconstruction. The electromyography performed on this patient 12 months post-surgery showed a return to normal femoral nerve conduction. Two (3.92%) patients developed superficial infections and were treated with parenteral antibiotics. No technical complications were encountered in any case. One (1.96%) patient had 120 degrees of flexion at the end of the rehabilitation, and although this patient could perform daily activities comfortably, they could not return to sports activities.

## Discussion

The most significant findings of this study were as follows: we observed that the desired clinical outcomes were achieved at a faster rate following the implementation of controlled accelerated rehabilitation in patients post-surgery. Additionally, we found no adverse clinical outcomes associated with the implants utilized in our study.

In ACL reconstructions, various materials have been utilized for graft fixation to the femur, and numerous biomechanical studies have been conducted on these materials. However, a definitive conclusion regarding the most suitable fixator for femoral fixation has not been reached [16-19]. Asık et al. achieved successful results using the cross-pin system and implementing accelerated rehabilitation [20]. Yosmaoğlu et al. compared Endobutton and transfemoral fixation methods and found no significant differences in muscle strength, motor coordination, or anterior tibial translation between the two groups [21]. In ACL reconstruction, graft fixation from the tibial side is referred to as the weakest part of the femur-graft-tibia structure. Therefore, in addition to tunnel fixation screws, the use of staples or screws is also frequently employed. Studies on the use of absorbable and metal screws within the tunnel have not found a significant difference in stability [22,23]. In our study, we used the Endobutton or cross-pin system for femoral fixation, and absorbable interference screws and staples for tibial fixation in all patients, and we did not encounter any implant failures. Similar to the literature, we did not observe a clinically significant difference between the two femoral fixation methods.

There is no consensus in the literature on the exact nature of accelerated rehabilitation. One common point is often the avoidance of brace usage, early initiation of loading, and early introduction of exercises that will lead to a full range of motion. The purpose of using a brace after ACL reconstruction is to protect the graft and ensure secure bone fixation of the implants. In various reviews examining the effects of the patellar tendon and hamstring tendon graft application and brace usage, brace usage did not have a positive effect on joint range of motion and graft stability [24-26]. Hence, we did not use a brace after ACL reconstruction surgery. Studies implementing accelerated rehabilitation often refer to the program described by Shelbourne et al. This program emphasizes specific preoperative and postoperative rehabilitation goals. While studies are limited, it has been noted that the return to social life and sports activities is expedited [27]. We implemented a modified version of the program described by Shelbourne et al., both before and after surgery. As we did not include patients undergoing meniscus repair in the study, we did not use braces postoperatively. We attributed the good range of motion in our patients and their early return to social life to the accelerated rehabilitation program we implemented.

This study has several limitations. First, it was conducted at a single hospital with a limited number of patients and short to medium-term outcomes, which may limit the generalizability of our results. Furthermore, the relatively limited number of patients prevented us from conducting a subgroup analysis. While our study yielded clinically successful outcomes, the most significant limitation was the absence of dynamometric measurements and reliance solely on clinical assessment tests for outcome evaluation. However, we believe that in the long term, this study will lead to multicenter prospective randomized comparative studies with larger sample sizes.

## Conclusions

In the controlled accelerated rehabilitation program applied after ACL reconstruction using hamstring autograft with femoral fixation by Endobutton and cross-pin, and tibial fixation by absorbable interference screw and staple, we clinically observed the durability of the implants. Patients returned to their daily activities earlier than anticipated before surgery. The controlled accelerated rehabilitation program can be safely implemented following ACL reconstruction.
